# Gait Analysis in a Box: A System Based on Magnetometer-Free IMUs or Clusters of Optical Markers with Automatic Event Detection

**DOI:** 10.3390/s20123338

**Published:** 2020-06-12

**Authors:** Javier Marín, Teresa Blanco, Juan de la Torre, José J. Marín

**Affiliations:** 1 IDERGO (Research and Development in Ergonomics), I3A (Instituto de Investigación en Ingeniería de Aragón), University of Zaragoza, C/Mariano Esquillor s/n, 50018 Zaragoza, Spain; 627471@unizar.es (J.d.l.T.); jjmarin@unizar.es (J.J.M.); 2 Department of Design and Manufacturing Engineering, University of Zaragoza, C/Mariano Esquillor s/n, 50018 Zaragoza, Spain; 3 HOWLab (Human Openware Research Lab), I3A (Instituto de Investigación en Ingeniería de Aragón), University of Zaragoza, C/Mariano Esquillor s/n, 50018 Zaragoza, Spain; tblanco@unizar.es; 4 Department of Musical, Plastic and Body Language Expression, University of Zaragoza, C/Pedro Cerbuna 12, 50009 Zaragoza, Spain; 5 GeoSpatiumLab Ltd., C/Carlos Marx 6, 50015 Zaragoza, Spain; 6 Department of Biomedical Engineering University of Zaragoza, C/Mariano Esquillor s/n, 50018 Zaragoza, Spain

**Keywords:** biomechanics, gait analysis, design, algorithm, gait events, applicability, reproducibility, minimal detectable change (MDC)

## Abstract

Gait analysis based on full-body motion capture technology (MoCap) can be used in rehabilitation to aid in decision making during treatments or therapies. In order to promote the use of MoCap gait analysis based on inertial measurement units (IMUs) or optical technology, it is necessary to overcome certain limitations, such as the need for magnetically controlled environments, which affect IMU systems, or the need for additional instrumentation to detect gait events, which affects IMUs and optical systems. We present a MoCap gait analysis system called Move Human Sensors (MH), which incorporates proposals to overcome both limitations and can be configured via magnetometer-free IMUs (MH-IMU) or clusters of optical markers (MH-OPT). Using a test–retest reliability experiment with thirty-three healthy subjects (20 men and 13 women, 21.7 ± 2.9 years), we determined the reproducibility of both configurations. The assessment confirmed that the proposals performed adequately and allowed us to establish usage considerations. This study aims to enhance gait analysis in daily clinical practice.

## 1. Introduction

Gait analysis based on full-body motion capture (MoCap) provides spatio-temporal and kinematic variables [1]. These variables are particularly useful for monitoring the progress of patients with musculoskeletal pathologies, and can offer ample possibilities in the rehabilitation field by assisting in decision making through measurement sessions before and after treatments, interventions, or therapies [2,3,4,5].

Gait analysis can be conducted using various types of MoCap technologies; two of the most common are optic-based and inertial measurement unit (IMU)-based technologies. Optical MoCap uses infrared light-emitting cameras situated in the room that can identify the position of spherical reflective markers placed on the subject. The reflective markers can be placed on the body either individually or grouped in clusters called rigid bodies (RBs) [6,7,8]. The IMUs are electronic devices that capture movement through signal processing of the output data of different built-in sensors (accelerometers, gyroscopes, and magnetometers) [9,10,11]. When optical systems use RBs, they have significant parallels with IMU systems, since the capture consists of associating an element (an RB or an IMU) with a body segment in both cases [12].

There are many systems for capturing human motion [13,14]; Marin et al. [12] reviewed commercially available IMU- and optic-based systems and analysed factors such as the devices used or their placement on the body. Other researchers, such as Tao et al. [15] and Muro-de-la-Herran et al. [16], reviewed existing approaches to analysing gait, including the use of algorithms to analyse gait patterns. These and other authors have discussed the potentialities and limitations of both technologies [6,7,8,9,10,11,12,13,14,15,16,17,18]. With regard to the potentialities, optical technology is characterised by its precise measurements; for this reason, it is considered the gold standard for MoCap systems. IMU technology is characterised by portability; as it does not require a fixed camera infrastructure, it can be used in different spaces in a relatively straightforward manner.

With regard to the limitations, some factors prevent the use and application of these technologies in daily clinical practice [12,19,20]. Amongst these factors, the measurement errors or inaccuracies that arise due to the following causes are important issues:

1. **Intrinsic variation:** Although the gait pattern is highly internalised in the brain, it is impossible to repeat the gait or any other movement in precisely the same way each time; there are always minimal individual variations, which are called intrinsic variations [21]. Intrinsic variation can be exacerbated if the experiment does not take place in a suitable environment that allows the subject to walk using his or her habitual gait pattern [22] or if the subject does not feel comfortable with the devices placed on the body. These situations can cause alterations in the movements [10] and can make the subject feel physically different, or uncoordinated [23].
2. **Soft tissue movements:** The movements of the skin, muscles, and other tissues around the bones are an artefact that occurs persistently in surface-marker MoCap systems [19]. The soft tissue effect is particularly notable on the thighs since the femur is covered by a considerable amount of tissue [24,25]. To avoid these effects, the optical systems based on individual markers often place the markers on bony structures, or “landmarks” [24]. However, it should be assumed that surface-marker MoCap systems do not represent the real movement of the bones [26].
3. **Relative movements between the device and the skin:** These relative movements are related to the adjustment of the fastenings that hold the devices on the body [25,27,28].
4. **Positioning:** It is difficult to position the devices in the same manner each time. The data obtained from acceleration and angular velocity differ from one location to another for the same body segment [27,29,30]. Differences in acceleration and angular velocity can be minimised by calibrating before the measurements are taken (see factor 7).
5. **Instrument accuracy:** The optical system’s accuracy is in the order of 1% [31] or 1 mm [19] for the measurement. IMUs use a sensor fusion algorithm to provide rotations from the signal of the built-in sensors [18,32]. IMUs are highly sensitive to disturbances in the Earth’s magnetic field, which disorients magnetometers, especially in indoor environments. In addition, a drift artefact is caused by cumulative gyro integration errors [33]. Thus, these types of sensors have accuracy ranges of 0.2°–1° (roll/pitch), 0.4°–2° (yaw) and a dynamic root mean square (RMS) of 1°–2° RMS, depending on the manufacturer [12].
6. **Gait event detection:** Gait events are relevant moments throughout the gait cycle, such as the initial or the final contact of each stride, which allow for the normalisation, superimposition, and analysis of the strides captured [15]. Events can be estimated using additional instrumentation (e.g., pressure platforms or instrumented templates), or using the movement data itself. In either case, there may be inaccuracies of a few milliseconds in the detection. In IMU-based systems, these errors in parameters such as the step length may be cumulative and translated as a few centimetres [34,35,36].
7. **Anatomical calibration:** When the devices are placed on the body, their coordinate systems always differ according to the anatomical segment on which they are fixed [18,37]. The anatomical calibration, also called sensor-to-segment alignment [38,39], is used to calculate the relative rotation between the device and the bone, which is assumed to be time-invariant once the sensor is mounted on the body [18,40]. The calibration allows for the calculation of the joints’ angles and the establishment of the participant’s neutral position, which corresponds to the zero rotation of all the body segments. Two main approaches [18,37] are used to accomplish the calibration: (1) Anatomical approaches, in which the user is asked to stay still in one or more body positions while the sensors are oriented to those expected in the static pose [41,42], and (2) functional approaches, in which the subject is asked to perform mono-dimensional or arbitrary motions to estimate the anatomical axes [39,43,44,45]. As it is not possible to adopt precisely the same position or to execute exactly the same movements in each measurement session, there is always intrinsic variation [21]. Similarly, anthropometric measurements of the participant are usually introduced during the anatomical calibration, which adds errors to those mentioned previously [40,41,44].

Given the errors and measurement inaccuracies exposed, it can be deduced that factors 1 and 2 are circumstantial and inherent in human biomechanics. However, factors 3–6 are related directly to the design of the product, the design of the experiment, or the design of the data processing. Factor 7—anatomical calibration—is a combination of both perspectives in that must be designed, but, at the same time, is related to intrinsic variation (factor 1). Therefore, it is relevant and necessary to make design proposals to improve factors 3 to 7, since such improvements will encourage the application of these technologies in daily clinical practice.

As a result of this reflection, we focused on two issues that have previously attracted interest in the literature; one is the development of IMU MoCap systems that are not affected or are less affected by alterations in the magnetic field, while the other is the development of MoCap systems (both optical and IMU) that detect gait events automatically using kinematic data without the need for additional instrumentation.

With regard to issue of the magnetic field, many studies have shown that it is possible to omit the magnetometer information [32,43,46,47,48]; however, there are two main drawbacks to the omission of magnetometers. The first is that there is no common horizontal reference (heading). The exposed anatomical calibration plays a key role in overcoming this problem, because this process deduces the heading direction [18,37]. In addition, recent research has revealed that the exploitation of kinematic constraints (such as boundary conditions in the degrees of freedom or the range of motion of the joints) can improve the heading calculation [45,46,49,50,51,52]. The second drawback is that, when the magnetometer information is omitted, the cumulative gyro integration errors are not corrected, which results in drift errors that increase with the capture time [33,53]. The most straightforward approach for resolving the drift errors is to limit the capture time, which is appropriate when only a short period is required to execute the movements being investigated [38]. For long-term captures, one approach is to use the aforementioned kinematic constraints, since they limit the drift artefact [45,46,49,50,51,52]; another possible method is to use zero-velocity updates [54] or dead reckoning [55] algorithms, which reset integration and acceleration errors when detecting zero-velocity periods during the gait.

With regard to detecting the gait events, numerous studies have proposed algorithms to detect gait events using kinematic data, both in IMU and in optic systems [34,35,36,56,57,58,59,60]. Algorithms usually search for events in the motion curves, such as the orientation, displacement, linear/angular velocity, or acceleration curves. Many computational methods have been used to search for these events, ranging from peak searching algorithms (e.g., [58]) to hidden Markov models (e.g., [61]).

Although there have been substantial contributions and achievements along these lines, it is necessary to discuss and propose complete solutions that overcome magnetic field alterations and detect gait events automatically [37]. The combination of these characteristics could enhance IMU and optical technologies in daily clinical practice.

When proposals are made along these lines—or along any others related to the errors collected—indicators and metrics are required to assess the quality and validity. In this regard, although each proposal may have individual indicators and metrics, reproducibility is the most general and important indicator that a MoCap gait analysis system should consider [19,28]. Satisfactory reproducibility results show that, under the same conditions, the system produces similar data every time it is used, and indicate that the system has sufficient precision to compare results, both within a subject over time and within groups of subjects, which is highly relevant for monitoring the progress of rehabilitation [62].

Moreover, reproducibility is an index that has value in itself. Reproducibility values can be used as a threshold in a capture comparison, allowing researchers to identify whether the change between two sessions is due simply to measurement errors and is thus not attributable to real changes in the subject [63]. In this regard, the minimal detectable change (MDC) index has been identified as one of the most relevant reproducibility indexes to judge the likelihood of real improvement (or impairment) in a subject [64,65,66,67,68].

In this study, we present a MoCap gait analysis system called Move Human Sensors (MH). This system can be configured using IMU technology (MH-IMU henceforth), or using optical technology (MH-OPT henceforth). The MH system includes two proposals: (1) an anatomical calibration procedure that allows for the deactivation of the IMUs’ magnetometers to avoid the magnetic influence, and (2) an algorithm that detects gait events from kinematic data without additional instrumentation.

We determined the reproducibility of both configurations via a test–retest reliability experiment with thirty-three healthy subjects. The study allowed us to evaluate the proposals of the MH system, to establish the usage considerations, and to compare the reproducibility of both configurations to each other and to similar studies. The concept of “gait analysis in a box”, which was inspired by Najafi et al. [69], appears in the title of the article to highlight the potential of these technologies, particularly IMU, to enhance the application of gait analysis in daily clinical practice.

## 2. Materials and Methods

In this study, we introduce the MH MoCap gait analysis system, which has two configurations, namely MH-IMU and MH-OPT. This section describes the MH system (Section 2.1), as well as the design of the experiment that we conducted to evaluate reproducibility (Section 2.2).

### 2.1. The Move Human Sensors (MH) System

The MH-IMU configuration uses up to 15 inertial sensors, specifically the NGIMU devices from x-io Technologies [70], which are calibrated by the manufacturer, and filters and processes the signal internally to transmit the rotations: in our case, quaternions.

The MH-OPT configuration uses up to 15 ad-hoc-designed rigid bodies (RBs), and a set of 12 cameras to capture the position and orientation of the RBs (OptiTrack Flex 13 cameras using Motive software [71]). Each RB is a cluster of three reflective markers (diameter 14 mm) placed on a rigid 3D-printed surface [6,7,8]. The markers for each RB have a unique spatial relationship (i.e., a unique marker distribution and a unique marker-to-marker distance) because this allows the software to differentiate one RB from another.

In the following sections, the MH system is presented in its full-body configuration (15 IMUs or RBs), although the system can be configured with fewer devices. In fact, in our reproducibility study, we configured the system with fewer devices because only the information from eight devices (IMUs or RBs) were needed (the feet, calves, thighs, pelvis, and chest) to analyse gait. 

With regard to the accuracy of both configurations, according to the manufacturer’s webpage, the NGIMU has an orientation accuracy of <1° RMS (pitch/roll) and <2° RMS (heading) [70]. The maximum orientation error $\left(E_{o}\right)$ of each RB is shown in Table 1. This error was calculated using the minimum marker-to-marker distance $\left(D_{min}\right)$ and the mean positioning error $\left(E_{p}=0.34\;\mathrm{mm}\right)\;$ of each marker provided by the Motive [71] software when the 12 cameras were calibrated.

**Figure 1:**
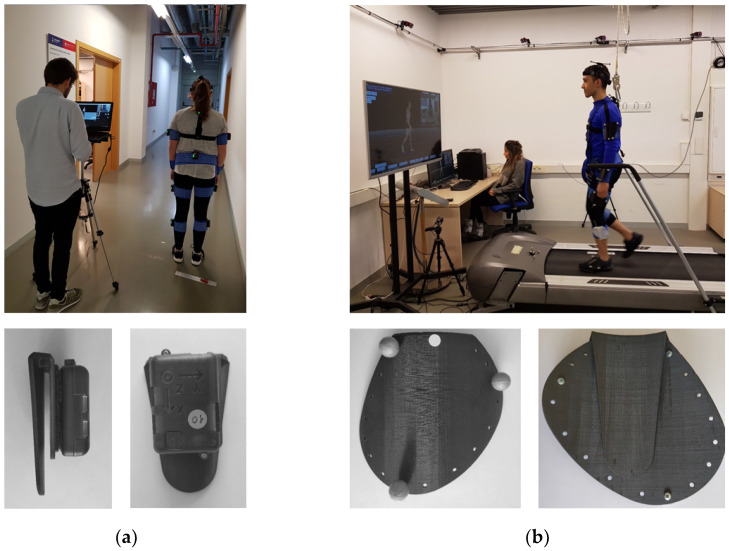
(**a**) Move Human Sensors (MH) system configured with inertial measurement units (IMUs) (MH-IMU); (**b**) MH system configured with optical technology (MH-OPT).

IMUs or RBs are placed on the body with elastic bands. We developed U-shaped, 3D-printed bases, one to place on one side of the U below the band, and the other to hold the IMU or the RB (Figure 1). Each base was designed individually to fit each body segment (adult’s anthropometry), and has small ribs on the inside of the U to guarantee rigidity and stability. The U-shape was chosen to facilitate the placement and decrease the preparation time. In this manner, the elastic bands are adjusted on the participant first to then position the bases with the IMUs or RBs; therefore, before completing a capture with one participant, the next participant may already have had another set of elastic bands put in place.

Figure 1 shows the MH-IMU configuration in which the subject walks on the floor and the MH-OPT configuration in which the subject walks on a treadmill (EXE T800 modified with the control panel placed independently). The MH system does not specify the use of the floor or a treadmill; however, we chose this disposition for either enhancing a specific advantage or for reducing a particular limitation of each configuration. The use of the floor in the MH-IMU configuration ensures its portability and makes measuring a realistic pattern possible. The treadmill in the MH-OPT configuration decreases the capture area and thus the number of cameras required; in addition, it allows for capturing numerous strides in standardised conditions (constant gait speed) without the subject needing to turn around [72,73,74].

It can be assured that the gait pattern will not be identical on the floor and on the treadmill. Although other studies have shown that the differences between both situations are small [74], we assumed that the gait pattern on the floor is one phenomenon and that the gait pattern on the treadmill is another. Therefore, in this study, we compared them solely in terms of reproducibility because this indicator is independent of the phenomenon being measured.

If required, the MH system can be configured to record live video with up to two Logitech C920 webcam cameras synchronised with the MoCap. Two cameras are used in the MH-OPT configuration, one in front of the subject in the direction in which he/she is walking, and the other at the side of the treadmill; the MH-IMU configuration uses one camera placed on the tripod that holds the computer.

With regard to communications, in the MH-IMU configuration, the devices are connected to the computer via Wi-Fi using the open sound control (OSC) communication protocol, and send the quaternions at a frequency of 60 Hz, which is sufficient to capture human movement at walking speed [75]. A portable router (Netgear Nighthawk M1) establishes the Wi-Fi network to which the computer and IMUs are connected at 5 GHz. In the MH-OPT configuration, the MH software is connected in real time via a virtual-reality peripheral network (VRPN) protocol to the Motive software [71], which transmits the transformation matrix of each RB at a frequency of 120 Hz.

The MH software, which integrates the mentioned features, was implemented using WorldViz-Vizard 6.2 (Python 2.7). As detailed in the following sections, the software captures the motion, transmits it to a human model in real time, and processes the information to detect the gait cycle and to generate variables.

#### 2.1.1. Human Model 

**Figure 2:**
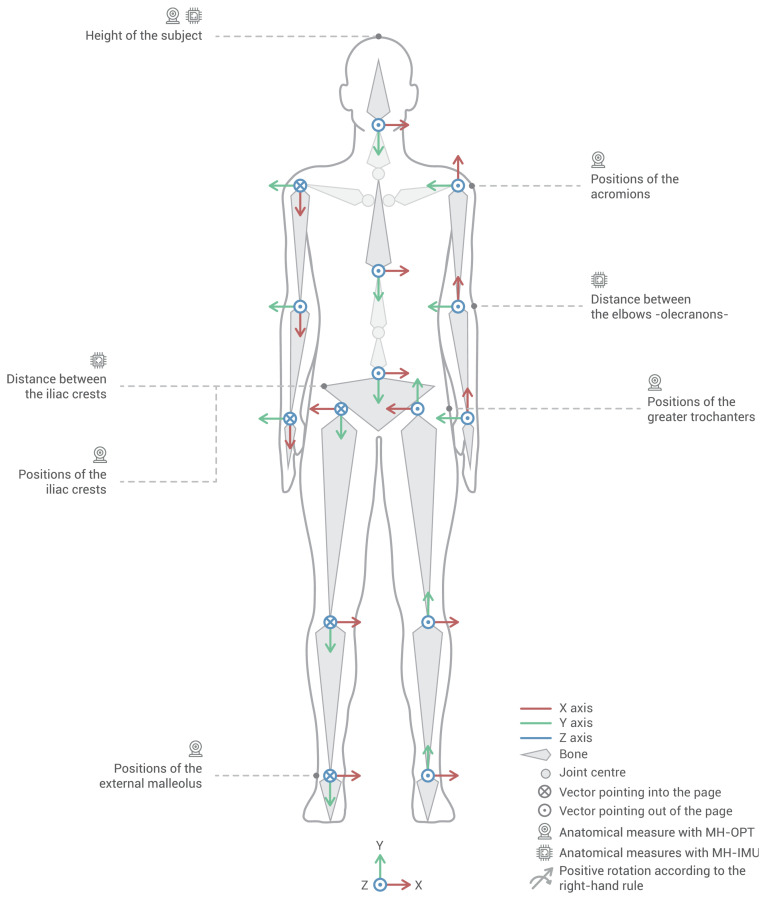
Human model in a neutral position, the local coordinate system for each bone, and the anatomical measurements needed. The positive rotation directions are interpreted according to the right-hand rule. Source: figure by the authors, icons (chip and camera) by Darius Dan and Vitaly Gorvachev from Flaticon [80].

The MH system uses the real-time motion information provided by the IMUs or RBs to animate a human model or avatar adjusted to the anthropometric dimensions of the participant. The human model was created using the ‘Genesis 2’ model of DAZ Studio 4.10 software [76]. In order to achieve a smooth real-time visual representation, the avatar mesh was reduced from 40,000 polygons in the original model to 7000 in our version. The resulting human model is a 20-bone skeleton, of which 15 bones are associated with the IMUs or RBs placed on the body. Figure 2 shows the human model in a neutral position and the local coordinate system for each bone. These coordinate systems are situated on the centres of the joints at the beginning of each bone (e.g., the centre of the femur bone is situated on the centre of the hip joint), except for the pelvic bone, where the centre is located at the geometric centre of the pelvis. The coordinate systems of the bones follow the right-hand rule for interpreting the directions of the bone rotation as positive or negative.

In order to adjust the length of the segments of the human model to the participant’s dimensions and to locate the centres of the joints, as shown in Figure 2, the rater needs to take specific anthropometric measurements. In the MH-IMU configuration, the rater must measure the height of the subject from which the human model is scaled, the distance between the elbows, from which the shoulder width is calculated and adjusted by projecting the angle of the arms in the frontal plane, and the distance between the iliac crests, from which the width of the hips is determined and adjusted (see Bell et al. [77]). In the MH-OPT configuration, the RB located on the head measures the height of the subject automatically; in addition, using an instrumented pointer synchronised with the MH software, the rater must measure the anatomical points (landmarks) of the acromions, greater trochanters, external malleolus, and iliac crests [78,79].

#### 2.1.2. Fitbody Calibration Process

**Figure 3:**
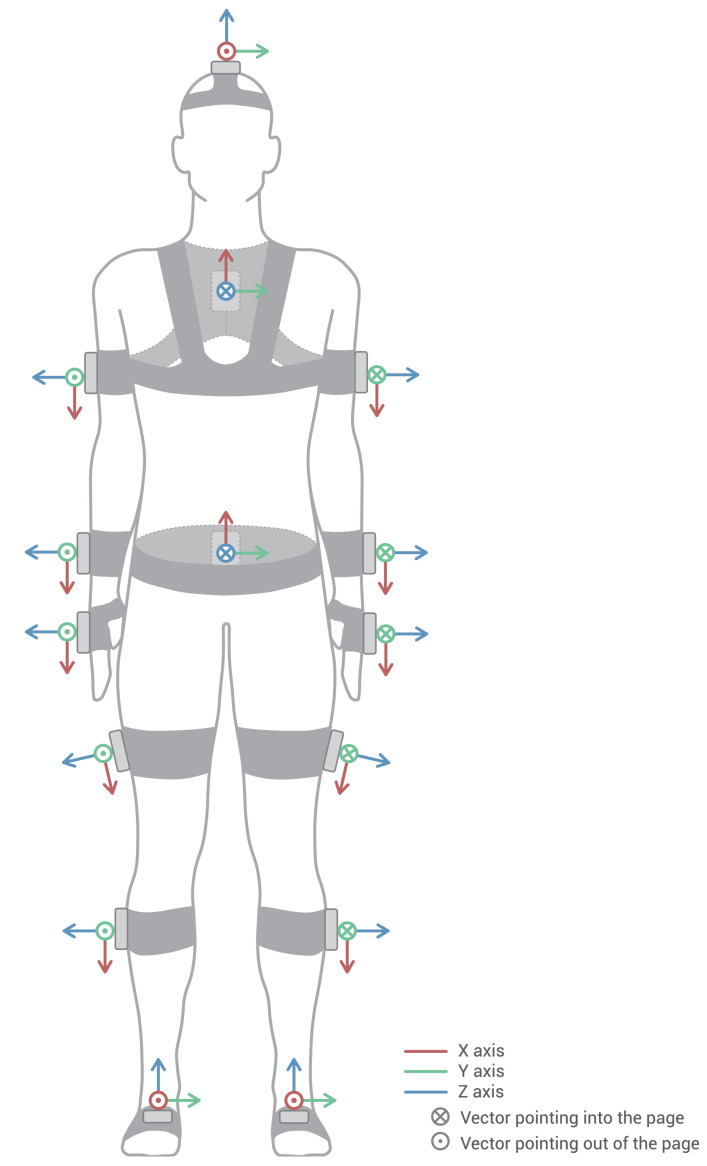
Placement of the devices on the body and the coordinate system for the devices. The IMUs and RBs are placed in the same positions because the surfaces that rest on the body are similar in size and shape.

Before conducting the gait capture, it is necessary to perform an ad-hoc calibration process, which we call Fitbody. This procedure includes a correction for magnetic north [81] (applied to MH-IMU) and an anatomical calibration [40,41,44] (applied to both MH-IMU and MH-OPT). As a result, this process establishes the participant’s neutral position and saves it for future reference. This position corresponds to the zero rotation of all the body segments, based on which each rotation will be positive or negative according to the established sign convention (Figure 2). Conceptually, the anatomical calibration is similar to the taring process of a scale in which we subtract the weight of a container, except that, in this case, we subtract the angles that the devices register on the surface of the musculature when the participant is in the neutral position. This process links IMUs or RBs to the human model’s bones virtually; in other words, it links the coordinate systems of the bones of the human model (Figure 2) to the coordinate system of each IMU or RB (Figure 3).

**Figure 4:**
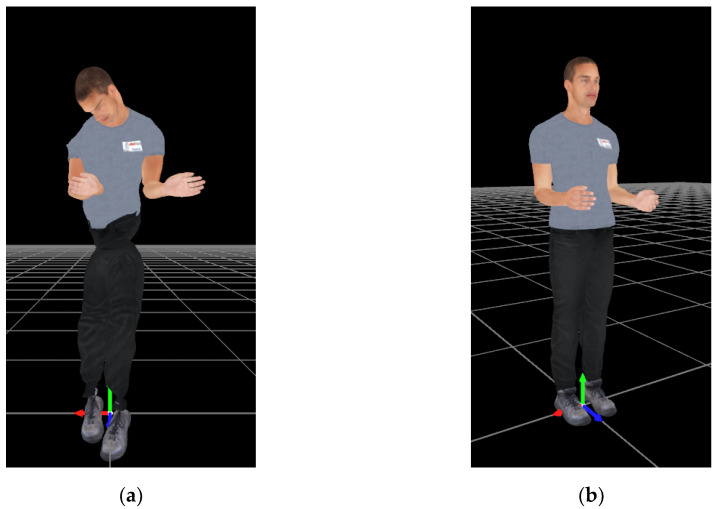
Human model (**a**) before and (**b**) after the Fitbody process in the MH-IMU configuration with the magnetometers disabled.

The Fitbody is required in both the MH-IMU and MH-OPT configurations. However, in the MH-IMU configuration, the magnetometers can be disabled as the Fitbody process infers the heading of the IMUs, thus avoiding the adverse effects that disturbances in the magnetic field may cause. In addition, as Lebleu et al. [38] explained, only a short time is required to capture the gait, and it is possible to repeat the Fitbody process before each capture, which is a sufficient procedure to overcome drift errors [33,53]. Figure 4 shows the Fitbody’s effect on the human model.

**Figure 5:**
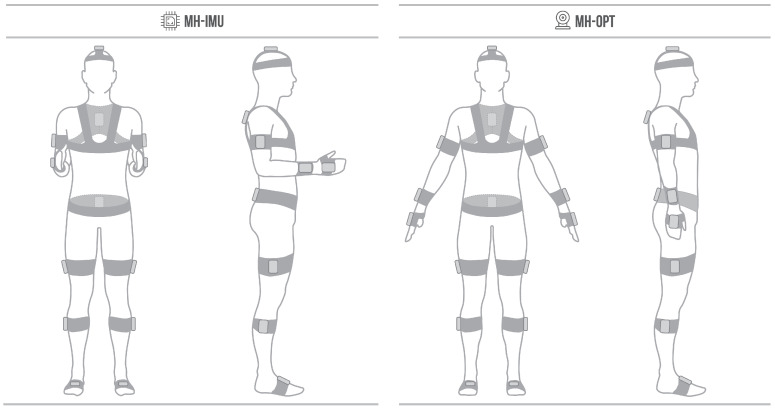
Fitbody position in the MH-IMU and MH-OPT configurations.

At the operational level, the calibration process requires the participant to adopt a specific static body position (Figure 5); at the same time, the rater needs to execute the Fitbody function implemented in the MH software. As mentioned, there are also “functional calibration” approaches in which the participants have to move the segments to determine the sensor-to-segment orientation. Although these functional procedures could be more precise than when remaining in a static pose [82], they are too demanding, particularly when several segments are considered, or when the system is intended to be used in pathological populations that may have substantial motion limitations [37].

**Figure 6:**
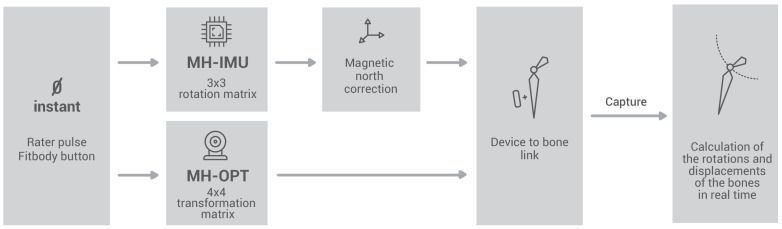
Fitbody calculation process.

The Fitbody calculation process is based on the scheme in Figure 6, in which we call the moment at which the rater launches the process instant 0. In the MH-IMU configuration, the quaternions read from the sensors are transformed into 3 × 3 rotation matrices (see “transformations”; Python library [83]); these matrices rotate the bones around the joints. In the MH-OPT configuration, the movements characterised by 4 × 4 transformation matrices read directly from the Motive software [71], including the rotations and displacements of the RBs. Therefore, RBs rotate the bones and displace them according to their centres of instantaneous rotation. Nevertheless, the term “rotation matrix” will be used in the explanation for both cases.

**Figure 7:**
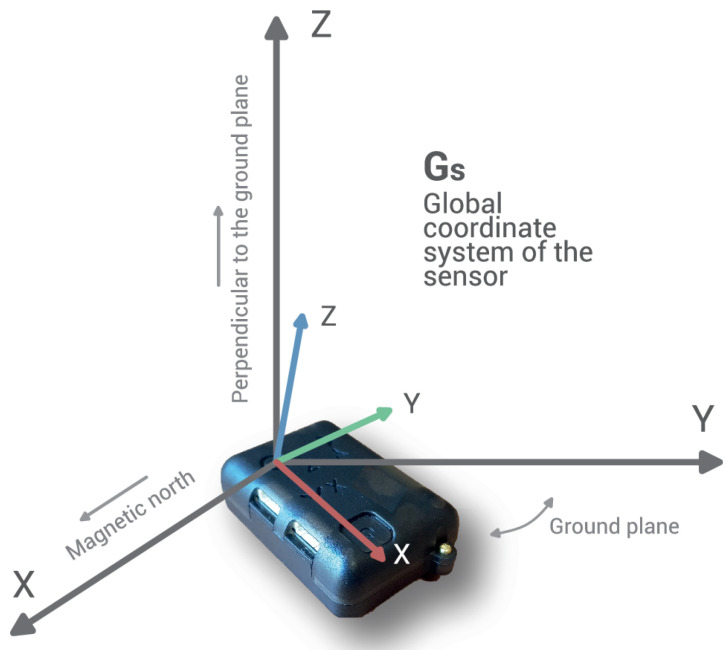
The global coordinate system of an IMU (Gs).

The first step in the MH-IMU configuration is to correct the magnetic north (heading) of each sensor. At instant 0, each IMU sensor has its own global coordinate system, which is called Gs (see Figure 7). In Gs, the *Z*-axis is perpendicular to the ground and coincides in all sensors, since the data are taken from the gravity measurement. The *X*-axis is at a right angle to the *Z*-axis (i.e., parallel to the ground). It corresponds to magnetic north when the magnetometer is activated, and to a random direction when the magnetometer is disabled. The *X*-axis is different in each sensor; there are significant differences in the absence of magnetometers and a few differences when magnetometers are present since, even when sensors are placed on the body (i.e., all the sensors are within <2 m), the measure of magnetic north is different in each location [81]. Finally, the Y-axis is at right angles to the X- and Z-axes in accordance with the right-hand rule.

Either with or without magnetometers, due to the *X*-axis disposition, it is necessary to establish a global coordinate system that is shared by all the IMUs, which is called G. The MH application establishes the Gs of the sensor located on the pelvis as G because it is the first element in the kinematic chain; in other words, MH copies the magnetic north of the sensor located on the pelvis to all the sensors.

The magnetic north correction is based on the assumption that, at instant 0, one of the axes of each sensor is placed on the body in a particular manner. This assumption is detailed in Table 2. The criterion for choosing the axis of each sensor is to identify the axes that is most parallel to the ground considering the natural inclination of the sensor when placed on the body. Note that the orientation of the sensors in other axes or their height on the body segment do not influence the process (e.g., the fact that the chest sensor has an inclination around the *Y*-axis due to the inclination of the area in which it is placed, vertebra D2–D3, has no effect).

Given this assumption, the next step is to calculate the relative angle (α) on the ground plane between the axes mentioned in Table 2. For example, if we consider the thigh sensor that is placed on the outer face of the thigh, the *Y*-axis projected onto the floor should be at 90° to the *Y*-axis projected from the pelvic sensor, which is placed on the sacrum. Thus, if the relative rotation were 83°, the angle to be corrected would be α = 7°. This α angle is transformed into the RGs0G  rotation matrix via Equation (1). According to the notation used in this study, RGs0G  is read as the rotation of Gs with regard to G at instant 0.

(1) RGs0G=(cos(α)0sin(α)010−sin(α)0cos(α))

Subsequently, this matrix is multiplied by Rs0Gs, the rotation read from the sensor (to be corrected) at instant 0 (Equation (2)), resulting in Rs0G, the rotation of the sensor with respect to G at instant 0.

(2) Rs0G=RGs0G · Rs0Gs

Note that the magnetic north correction does not need to be applied in the MH-OPT configuration because the global coordinate system of each RB coincides with the global coordinate system of the world (G) and the rotation Rs0G of each RB is known.

The next step in the Fitbody process is performed in both the MH-IMU and MH-OPT configurations, and consists of linking the coordinate system of each bone in the human model (Figure 2) to the coordinate system of each device (IMU or RB) placed on the body (Figure 3). Equation (3) must be applied to link these parts:

(3) Rs0b=[Rb0G]T · Rs0G,

where Rs0b is the sensor rotation with respect to the bone at instant 0; Rb0G is the bone rotation in the Fitbody position, which is a known orientation; and Rs0G is either obtained from Equation (2) (MH-IMU) or is read directly from RB (MH-OPT). It should be noted that Rs0b is considered constant throughout the capture, since it is assumed that the sensor does not move from the segment to which it is attached, a hypothesis that, as explained in the discussion, is necessary to ensure sufficient attachments to the body.

After these steps, it is possible to calculate, in both configurations, the absolute or relative bone rotations during the rest of instant *i* of the capture. The rotation of each bone with respect to *G* (namely absolute rotation) can be identified using Equation (4).

(4) RbiG=RsiG · [Rs0b]T 

Similarly, Equation (5) enables the identification of the relative rotation of the joints; that is, the rotation of each bone relative to its parent bone (*p*). In our case, the hierarchy of the bones of the kinematic chain begins in the pelvis, which is the main bone; the pelvis is the parent of the thorax and thighs, the thighs are the parents of the calves, the calves are the parents of the feet, and so on.

(5) Rbip=[RpiG]T · RbiG

To identify the rotations in each plane, the rotation/transformation matrix of each bone is transformed into Euler angles, with the order of *X-*, *Z*-, and *Y*-axis of the human model (Figure 2). To accomplish this, we use the transformations Python library [83] with the “rxzy” order, where r means “rotating frame”. Thus, in the femur bone, the *X*-axis rotation represents the flexion–extension, the *Z*-axis rotation the abduction–adduction, and the *Y*-axis rotation the internal–external rotation.

In the MM-OPT configuration, each bone’s RbiG  transformation matrix includes the displacement of the bone’s centre with respect to *G* (i.e., the centre of the bone moves according to the movement of the RB to which it is linked). In the MH-IMU configuration, the displacement of each bone’s centre is calculated by direct kinematics [84] by using the joint rotations (i.e., Rbip)  and the length of each bone [84,85]. As mentioned, and as can be seen in Figure 2, each bone’s centre is located at the beginning of the bone, just on the joint with the previous bone of the kinematic chain. Thus, the displacements of the bones’ centres coincide with the displacements of the joints’ centres.

#### 2.1.3. Gait Event Detection Algorithm

**Figure 8:**
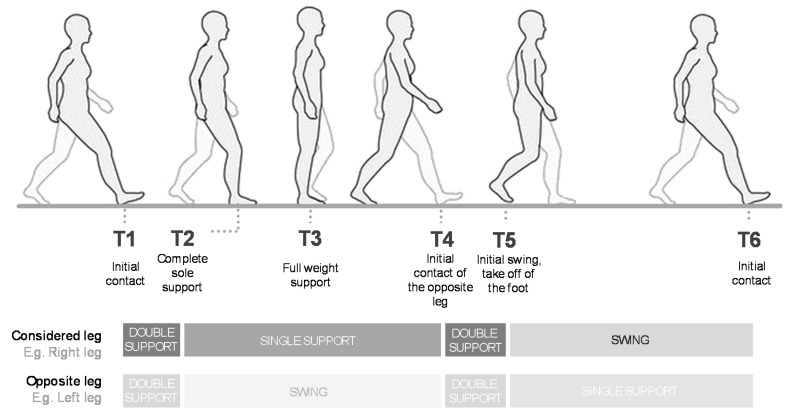
The gait events considered.

Once the gait capture has been completed, the walking pattern is analysed throughout the gait cycle, which is characterised by several key moments called gait events. These events delimit the start and end of each stride, identify the gait phases, and allow for the overlap and normalisation of movement curves from 0% to 100% [15,34,35,36]. The MH system identifies six gait events (T1–T6; see Figure 8). At this point, we differentiate between two important terms related to the gait cycle: the step length (of a specific leg), which is the distance between the centres of both ankle joints in the sagittal plane at T1, and stride length (of a specific leg), which is the distance between the position of the centre of the ankle joint at T1 and its position at T6 in the sagittal plane (in other words, the entire path of the centre of the ankle joint during the gait cycle).

**Figure 9:**
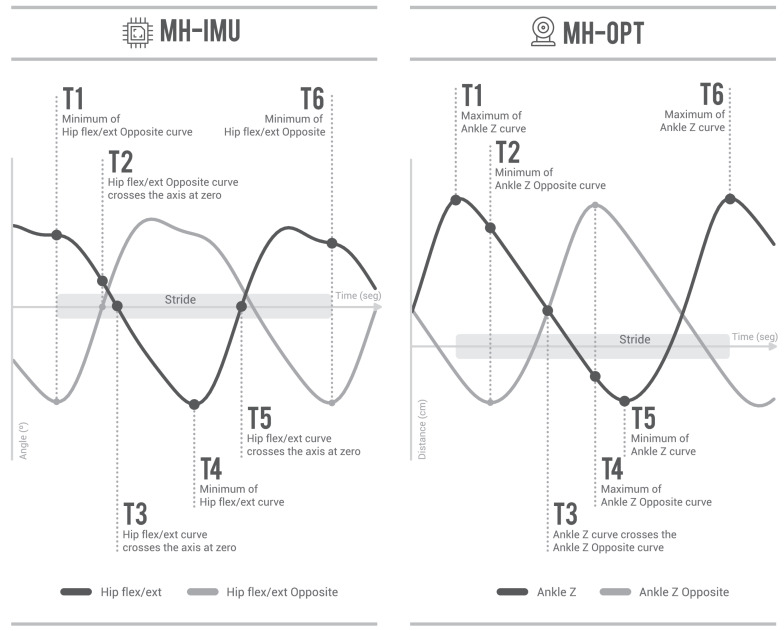
Logical rules for the detection of gait events in MH-IMU and MH-OPT configurations. To detect gait events, the MH-IMU uses the hip flexo-extension curve of both legs, and the MH-OPT uses the displacement of the centres of the ankle joints on the *Z*-axis.

The MH system uses specific movement curves and rules to detect gait events. Figure 9 shows the curves and rules established for each configuration. The algorithm was initially developed based on the researchers’ experience, and was then tested and improved iteratively using different gait captures from previous, unpublished experiments. These gait captures were of diverse participants, ranging from healthy subjects to individuals with valgus foot, osteoarthritis of the hip, ankle injuries, knee injuries, and non-severe spasticity. This iterative process was possible because the MH system allows for the visualisation of the human model’s movements and live video images from the synchronised cameras, and because the software can be configured to illuminate a sphere each time an event is detected.

The detection of gait events in the MH-IMU configuration is based on the hip flexion–extension curve and the same curve in the opposite leg. In the MH-OPT configuration, the detection is based on the curve of the absolute displacement of the centre of the ankle joint on the *Z*-axis, and the same curve in the opposite foot. In order to detect events in the MH-IMU configuration, the rater must select the sections to analyse; that is, the sections in which the patient walks in a straight line, excluding turns, starts, and stops. This operation is not required in the MH-OPT configuration because the gait capture is continuous, and does not entail stops or changes in direction. 

As seen in Figure 9, the rules for detecting gait events are based on search maximums and minimums of the mentioned curves. To avoid detecting false maximums or minimums, we used a process to smooth these curves, specifically a “sliding window” method based on the convolution function in NumPy (see “smoothing of a 1D signal” in the SciPy library [86]), with a window size of 0.2 s.

To justify the use of the hip flexion–extension in the MH-IMU configuration, it should be noted that the rotation matrices of the IMUs rotate the bones in relation to the joints, and their displacements depend on the lengths and angles in the kinematic chain. If it were to use the displacement of the ankles’ centres on the *Z*-axis (as in the MH-OPT configuration), this measurement would be obtained from the sum of the angles in the kinematic chain, and would involve the accumulation of errors from different sensors and different anthropometric measurements. Therefore, it is more reasonable to estimate the gait events using the hip flexion–extension curves, which are only influenced by the pelvic and the thigh sensor errors, and not by the entire kinematic chain. Conversely, in the MH-OPT configuration, the use of the displacement of the ankle joints’ centres on the *Z*-axis is justified because each bone moves and rotates driven by the RBs transformation matrices; therefore, each RB has independent precision. This independence makes the use of the ankles’ centres to estimate gait events, whereby the entire path of the feet can be appreciated, a reasonable choice.

### 2.2. Test–Retest Study Design

The reproducibility of the MH-IMU and MH-OPT configurations was studied via a test–retest reliability study with thirty-three healthy subjects (20 men and 13 women; 21.7 ± 2.9 years of age; height 173.1 ± 9.1 cm; weight 66.9 ± 11.0 kg). All participants met the inclusion criteria of being over eighteen years of age, being able to walk unaided, not having injuries or having undergone recent surgeries that limited the mobility of the lower limbs, not having received recent pharmacological treatments, and not having had regular incidents of vertigo or dizziness. The study was approved by the Bioethics Committee of Aragón, Spain (N° 12/2018), and informed consent was obtained from all the participants.

**Figure 10:**
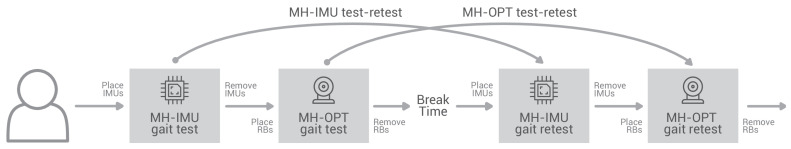
Sequence followed by the participants in the test–retest reliability experiment (begun randomly with MH-IMU or MH-OPT).

The test–retest reliability study consisted of repeating the gait test under the same conditions following the process in Figure 10. The devices placed on the body were removed between the tests, the rater did not change, and the MH-IMU or MH-OPT tests started randomly. Three hours elapsed between the test and the retest; during this time, we ensured that the participants did not perform any activity that could influence the retest (e.g., physical exercise, eating a big meal, and so forth); they remained in the laboratory participating in other nonphysical tests and/or sat in an adjoining study room. The three-hour period was chosen because these reproducibility results can be particularly useful for assessing changes in the interventions applied during the same session or on the same day.

In the MH-IMU configuration, the participant walked naturally in a straight line at a self-selected speed for six metres, then turned around and walked back multiple times. To become familiar with the instrumentation, the subjects walked along the path for five minutes; they then stopped to conduct the Fitbody process. This stop was necessary due to the aforementioned limited duration of the Fitbody process without magnetometers. Subsequently, the participants continued to walk until they had reached 25 strides (i.e., 25 complete gait cycles, or 50 steps). To count the 25 strides, only those performed in a straight line were considered, and turns, starts, and stops were excluded. The rater observed the participants and questioned them to confirm that there were no signs of fatigue.

In the MH-OPT configuration, the participants first conducted the Fitbody; then, they became familiar with the instrumentation by walking on a treadmill for five to ten minutes [72]; finally, we measured the 25 strides. The treadmill speed was stipulated, as described by England and Granata [87], according to the theoretical natural gait speed calculated using the leg length (distance from the centre of the hip to the centre of the ankle) of each subject. England and Granata’s [87] formulation is included in the MH software, and the mean of the length of both legs is computed automatically from the anthropometric measurements. The same speed was used in the test and in the retest. The rater asked each participant to confirm that it was a comfortable speed, and that he/she did not experience fatigue; this procedure meant that it was not necessary to modify the theoretical natural gait speed, and any participant reported or showed fatigue in any configuration.

The 25 straight strides measured in each test were chosen according to Kribus-Shmiel et al. [22], who assured that statistical stability and normality were achieved within 23 strides, and that this number of strides was sufficient to represent the mean behaviour in gait analysis.

The data analysis was integrated into the MH software to obtain the spatio-temporal and kinematic variables (Table 3). The kinematic variables were the ranges of movement between two gait events, and their sign of rotation (positive or negative) was interpreted according to the right-hand rule, as shown in Figure 2.

The values of the 25 strides in each test were averaged to consider the variability of the gait [21]. Once the data from the thirty-three subjects had been collected, the MDC index at 95% confidence (MDC95) was calculated using Equation (6) [64,65,66,67,68],

(6) $$\mathrm{MDC}95=1.96\;\sqrt{2}\;\mathrm{SEM};\;\mathrm{SEM}={\mathrm{SD}}_{pooled}\;\sqrt{1-\mathrm{ICC}}\;,$$

where SD is the *pooled* average of the standard deviation in the test and the retest, ICC is the intraclass correlation coefficient, and the SEM is the standard error of the measurement.

The effect size of the MDC95 index was calculated as dimensionless using Equation (7), resulting in MDCes95, which indicates the number of standard deviations the experiment is capable of detecting [88],

(7) MDCes95=MDC95/SDtst ,

where *SDtst* is the standard deviation of the initial test.

## 3. Results

Table 4 shows the results obtained in the test–retest reliability study using MH-IMU and MH-OPT configurations. This table includes the mean differences between the subject’s tests (i.e., the mean of the differences between the test and retest of each subject) and the reproducibility, which is represented by ICC, MDC95, and MDCes95 indices. The variables described previously in Table 3 were calculated for the right-hand (R) and for the left-hand (L) sides.

**Figure 11:**
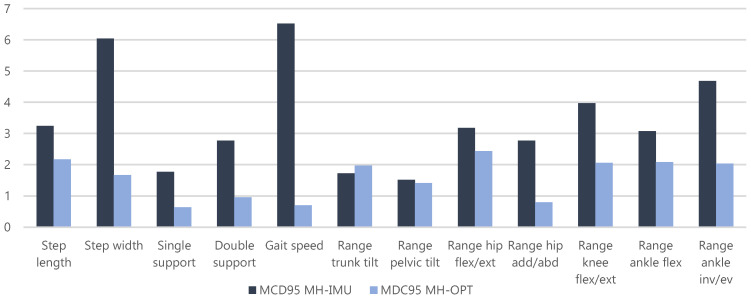
MDC95 results for both configurations averaging the right- and left-hand sides.

The gait of healthy subjects is usually symmetrical, with minor deviations [89]; in our case, the average difference between the right- and left-hand sides was 1.0 cm for step measures (*step length* and, 0.3% for support percentages ( and *double support)*, and 0.5° for kinematic measures. These results were notably less than the MDC95 magnitudes; therefore, the right- and left-hand sides were averaged, and the results are shown in Figure 11.

## 4. Discussion

In this study, we presented the MH MoCap gait analysis system, which has two configurations—MH-IMU and MH-OPT. The MH system includes two key proposals, which are (1) an anatomical calibration procedure that permits the deactivation of the IMUs’ magnetometers to avoid magnetic influence, and (2) an algorithm that detects gait events from kinematic data without additional instrumentation.

These proposals add value to the field of gait analysis, are applicable to other systems, and overcome specific barriers, such as the need for a magnetically controlled environment, which affects the operation of IMU technology, and the need for additional instrumentation or a laborious data analysis process to detect gait events, which affects the IMU and optic technology operations. This study posits a reduction in the time and resources dedicated to each patient during gait analysis, and promotes the application of both technologies in daily clinical practice.

In this section, we discuss the reproducibility of the results obtained in the experiment (Section 4.1), as well as the considerations and limitations of the MH system, particularly those related to the proposals that the system incorporates (Section 4.2).

### 4.1. Discussion of the Test–Retest Results

The test–retest reliability experiment verified the general operation of the proposals described. This experiment proved the reproducibility of both configurations. Reproducibility is known as the most general and important indicator that MoCap gait analysis applications should consider [19,28]. The average MDC95 results for the MH-IMU configuration were 4.6 cm for the step measures, 2.3% for the support percentages, 6.5 cm/s for the gait speed, and 3.0° for the kinematic variables. The results for the MH-OPT configuration were 1.9 cm, 0.8%, 0.7 cm/s, and 1.8°, respectively. The results were similar for both configurations, although they were slightly better for the MH-OPT configuration, which could be justified by the greater precision of this technology [6,7]. A greater difference was observed in the gait speed; this was expected because the speed on the treadmill did not change between the test and the retest [72,73].

Furthermore, in the MH-IMU configuration, the average MDCes95 was 0.86, and was 0.74 in the MH-OPT configuration; thus, according to Hopkins et al.’s [90] classification, it can be confirmed that both configurations can detect between “moderate” and “large” changes. In terms of ICC, the MH-IMU had an average of 0.90 and the MH-OPT of 0.93; according to Koo and Li’s [91] classification, these results show “excellent” reproducibility. Therefore, we can assert that the reproducibility was satisfactory; thus, the proposals performed adequately at a global level.

With regard to the application of the results, it should be mentioned that the MDC95 is usually defined as the minimal amount of change within a subject that is not due to a random variation or an error in measurement [65]. This definition establishes an interesting framework in which to apply the MH system in daily clinical practice. As described by Marin et al. [20], the gait test could be used as a medical test based on pre- and post-measurement sessions of the rehabilitation treatments applied. The reports generated would show the changes that occurred in each patient’s gait between the pre- and post-sessions. These reports could be used to make decisions about treatment (such as to continue treatment, to change treatments, to increase the intensity of treatment, and so on). In this context, it is necessary to consider the approximate magnitude of the expected changes. For example, if the intention is to evaluate a plantar orthosis for a slightly valgus foot, the changes in the subject’s gait pattern are expected to be small; moreover, these changes will be highly concentrated in the foot, possibly with minor effects in the knee and hip; therefore, an accurate MDC95 will be necessary. Conversely, if the aim is to evaluate the impact of a drug designed to treat a severe vertiginous pathology, it is expected that pronounced changes to the entire body will be seen; therefore, a less accurate MDC95 will be sufficient.

In addition, it is relevant to mention that the MDC95 was conducted following a three-hour break between the test and the retest, which can be particularly useful for assessing changes in the interventions employed during the same session. For example, two successive gait analysis tests could be used to select the optimum torque to be exerted by the mechanical knee joint of a transfemoral prosthesis; the first test could be conducted using the pre-established or usual torque configuration, and the second using a configuration that is predicted to be superior. Such an experiment could confirm or reject an orthopaedic technician’s hypothesis regarding the optimal torque for a specific patient.

Continuing with the application of the MDC95 results, if changes in groups of subjects need to be evaluated, the revealed MDC95 values should be modified according to Equation (8), in which *n* is the sample of subjects to be evaluated [68,92].

(8) $$MDC95_{group}=\frac{MDC95}{\sqrt{n}}$$

In addition, it is important to compare the results to those in other studies (see Table 5). The following criteria were used to select similar studies: (1) experiments that involved the subjects walking either on the floor or on a treadmill, (2) healthy subjects, (3) young subjects, and (4) studies that provided the MDC value. It should be noted that it was necessary to average some of the values in our study and in the other studies (see the row of “notes” for Table 5) in order to provide comparable values.

The results of the other studies can be grouped according to different criteria; for example, according to the technology used (optical/IMU), or according to the experimental conditions (floor/treadmill). However, to obtain a general conclusion regarding reproducibility, and due to the limited number of studies, it can be summarised that the average MDCs in other studies were 5 cm for the step measures, 1.7% for the support percentages, 12.7 cm/s for the *gait speed*, and 4.6° for the kinematic variables. Thus, from a general perspective, we can confirm that the reproducibility of both configurations was better than the average found in the literature. Nevertheless, as presented in Table 3, certain values in our study showed worse MDCs than were found in other studies (see asterisks in Table 5); of these values, the only one with a reasonable difference in magnitude was the *step width* in the MH-IMU configuration. We consider that this result is justified because the data for this variable in other studies were derived from optical systems and, as described, these systems achieve more precision than do IMU-based systems in a bone as distal as the foot, where the *step width* is computed.

Nonetheless, we compared the results with caution. We attempted to find the maximum similarity to other studies by using the aforementioned criteria. However, these studies used different anatomical calibration procedures, different parameters and, in particular, different times between the test and the retest, which were longer in the collected studies than in this study. All these differences may explain our superior results.

To discuss the time between captures, we should return to the factors mentioned in the introduction. It can be stated that 1—intrinsic variation and 7—anatomical calibration factors depended on the time that had elapsed between captures. However, as long as the devices were removed from the subject between the tests, the experiment fully considered the errors derived from factor 2—soft tissue movements, 3—relative movements between the device and the skin, 4—positioning, 5—instrument accuracy, and 6—gait event precision. The time dependency of factors 1 and 7 is related to the limited human ability to remember unconscious body control sensations. Although the instructions given to participants were relatively simple (walk at a natural pace in a straight line, or on the treadmill), when a long time has elapsed, one cannot remember these unconscious body control sensations accurately, such as the exact placement of the body for the Fitbody position (such as how stretched one was, whether the muscles were relaxed or not, and so on) or, for example, the level of control or strength applied in the impulse of each step. Thus, time has an influence and may have contributed to our superior results, but we could not determine the precise extent of this contribution.

To conclude the discussion of the results, it should be mentioned that we compared the MH-IMU and MH-OPT configurations solely in terms of reproducibility. Nevertheless, the results in Table 4 show that there are variables that exhibit notable differences between walking on a treadmill (MH-OPT) and walking on the floor (MH-IMU) that are even higher than the MDC values (step width, double support, range of trunk tilt, range of knee flexo-extension, and range of ankle dorsi/plantar flexion). Although other studies have found that there were small differences between walking on a treadmill and walking on the floor [74], our results suggest that we cannot consider both situations to constitute exactly the same phenomenon.

### 4.2. Usage Considerations and Limitations of the MH System

After discussing the results of the test–retest reliability experiment, it is necessary to identify and discuss the usage considerations, or precautions, and the limitations of the MH system, particularly the main proposals that the system includes.

Firstly, it is important to note that a full-body MoCap of 15 IMUs or RBs was presented. However, the system can be configured with fewer sensors depending on which kinematics are needed. The pelvis is always required, and IMUs or RBs must be added following the kinematic chain towards the upper and/or the lower body. In our case, only eight elements were necessary to analyse the gait. This number of devices decreases the preparation time and, in the MH-IMU configuration, reduces the cost considerably. At this point, it should also be noted that the main difference in the MH-IMU configuration from systems based on a single IMU (usually situated on the pelvis; e.g., [98]) or two IMUs (usually located on the feet; e.g., [92]) is that the angle of each joint is calculated in addition to the calculation of the spatio-temporal variables, which is relevant information for analysing gait patterns. With regard to reducing the number of devices, recent research has revealed that it is possible to apply inverse kinematics to calculate the movement of unequipped body segments (e.g., using the IMUs on the thigh and the foot to calculate the movements of the calf); this approach has been called “sparse inertial motion tracking” [45].

With reference to the Fitbody calibration process, we should discuss the north correction of the IMU sensors. The magnetic north correction should be applied either with or without magnetometers. Nevertheless, it should be noted that, if magnetometers are used, the correction has an important limitation. The corrected angle is based on the between-sensor differences in the measurement of magnetic north at the initial instant, but these between-sensor differences rarely remain constant when the subject is walking, and the IMUs can become disoriented. Conversely, when the magnetometers are disabled (as in our experiment), the between-sensor differences remain constant, which is an important advantage that allows for the use of the system in any environment.

However, disabling the magnetometers is not without limitations; due to the internal IMU sensor fusion algorithm, there is a drift error that increases with time in the absence of magnetic information, and the duration of the calibration depends on the integration of the drift of the gyros [33]. In our case, conducting a Fitbody process before each gait test allowed for sufficient time to obtain results that had satisfactory reproducibility. Thus, we conclude that the Fitbody process in the MH-IMU configuration with magnetometers disabled can be used to capture data for short-term gait analysis, but could lose precision in longer captures. The average duration of the 33 × 2 captures with the MH-IMU configuration was 39.4 ± 6.7 s. It would be useful to conduct future studies to evaluate the loss of precision over time.

Different approaches could be used to extend the capture time in the MH-IMU configuration. One is the exploitation of kinematic constraints; for example, using the knowledge that the elbow joint does not permit abduction/adduction, or that the shoulder cannot attain more than 180 degrees of abduction or flexion [45,46,49,50,51,52]. Another approach could be to use zero-velocity updates [54] or dead reckoning [55] algorithms that reset the errors when detecting zero-velocity periods during the gait. If the capture time were prolonged, both the MH-IMU and the MH-OPT configurations could be used for the for rehabilitation itself. As Georgiou [99] and Braga et al. [100] demonstrated, these types of systems can provide visual, auditory, and haptic feedback to improve gait patterns.

Continuing with the Fitbody process, it should be noted that, in both the MH-IMU and MH-OPT configurations, the position adopted by the participant (Figure 5) at the moment of executing the Fitbody function is an important issue. Due to the second step in the Fitbody process (the device-to-bone link), this position will be the neutral reference for the data capture, and the recorded angles will rely on this position. If a back flexion position were adopted by the subject in the Fitbody, when the subject adopted his or her neutral position, the human model would show an excessive back extension that would not represent reality. Therefore, the quality of the data captured is conditional upon the rater’s ability to instruct the participant to adopt the correct body position.

The rater has to memorise specific instructions and must be able to determine the participant’s neutral position, which depends on the participant’s anatomy and pathology. In our study, standing directly in front of the participant in the Fitbody posture and asking the participant to copy the pose was useful. Moreover, specific phrases such as “Now I need you to be very still, as in a picture”, “Place yourself in a neutral position”, “Face forward”, and “Place your feet parallel and at the width of your hips” were useful. In the MH-IMU configuration, “Place your arms at ninety degrees of flexion with the palms facing each other” was required, while “Place your arms outstretched at an intermediate height with the palms facing the body” was the requirement in the MH-OPT configuration.

Another important concern in the Fitbody function is the assumption that the devices are fixed securely [10] and positioned appropriately on the body [30], which is not a simple task [12].

With regard to fixing the devices to the body, the following guidelines assisted us to improve the process of attaching the devices. The straps that connect the devices to the body need to be sufficiently tight to prevent them from moving during the capture, they must respect the joint mobility space, and the participants must be able to reach their maximum ranges without discomfort or impediments [23]. It is necessary to ask the participant if he or she is comfortable and to modify the positioning or tighten the straps if necessary. If the subject is not comfortable wearing the devices, he or she may not move naturally [10], and may feel physically different, awkward, or uncoordinated [23]. Thus, the participant should walk and move around several times while employing wide ranges of movement; this ensures that the devices do not move significantly on the body.

With regard to the positioning of the devices on the body, certain considerations were particularly relevant. In the MH-IMU configuration, as shown in Table 2, the general criterion was to place one of the sensor’s axes in a particular direction on the body. Useful ways of accomplishing this were to verify that the leg and arm sensors were situated on the lateral surfaces of these extremities, that the *X*-axes of the chest and the pelvic sensors were aligned with the spine, and that the X-axes of the head sensor and the feet sensors were aligned with the direction in which the subject was pointing. In the verification process, the height of the sensor along the body segment to which it is attached and its direction with regard to other axes are irrelevant; these parameters depend on the subject’s anatomy and do not affect the Fitbody process.

If these positioning guides are not applied, the human model’s movements will not represent the real movement of the subject. For example, if we were to place the tight-fitting sensor on the lateral surface of the leg, but the *Y*-axis was not orthogonal to the *Y*-axis of the pelvis (20 degrees), the magnetic north correction made on the Fitbody would be incorrect; therefore, when the participant moved the hip on the sagittal plane, the leg of the human model would move on a plane rotated from the sagittal plane (i.e., 20 degrees).

The positioning guidelines in the MH-OPT configuration were derived from the adjustment of the human model’s joint centres. The pelvis and thorax RBs must be centred on the spine, the first in the sacrum area and the second on the D2 vertebra. The leg and arm RBs have to be fixed on the lateral surfaces of these extremities to make them more visible to the cameras. Finally, the feet RBs have to be fixed to the centre of the upper surface of the foot, aligning the sensor’s *X*-axis with the bone’s *Z*-axis. If the positioning is inadequate, the main implication will be that the human model’s joint centres will be positioned imprecisely, and the model will adopt a shape that will not correspond to the shape and dimensions of the subject.

With regard to the detection of the gait event, it should be noted that, as described, the proposed algorithm is reproducible. Nevertheless, further studies could be conducted to calculate the absolute accuracy of this algorithm in frame units. Absolute accuracy is usually obtained by matching the gait events detected by the algorithm to the events detected by one or more raters observing the live video recordings [34,35,36,56,57,58,59,60].

Similarly, it should be noted that the algorithm could be used for pathological gaits if the movement fulfils certain conditions. As described in Section 2.1.3, the event detection is based on the hip flexo/extension curves (MH-IMU configuration) and the displacement of the centres of the ankle joints on the sagittal plane (MH-OPT configuration). For this reason, it is necessary that the gait pattern shows a minimum of leg extension and flexion. If one of the legs were not to have any flexion or extension due to a pathology, it would not be possible to detect the events. Based on the same rationale, another factor that can have a negative effect is the analysis of a gait with considerable leg tremors in the sagittal plane; that is, forward and backward leg movements. As described, the signal is smoothed, but the algorithm may not detect the peaks correctly if such tremors are too great. Therefore, further research is required to test this algorithm in pathological populations.

To conclude the discussion section, it can be stated that this study presents a complete solution for gait analysis that can be used with satisfactory reproducibility, and which includes valuable proposals to enhance gait analysis in daily clinical practice. However, the MH system and the proposals that it includes are not without limitations; thus, the reflections provided in this section can be useful to improve applicability and to establish avenues for future research.

## 5. Conclusions

In this study, we presented the MH MoCap gait analysis system, which can be configured with magnetometer-free IMUs (MH-IMU) or with clusters of optical markers (MH-OPT). The MH system incorporates an anatomical calibration procedure that allows for the deactivation of the IMUs’ magnetometers to avoid the magnetic influence, and an algorithm that detects gait events from kinematic data without additional instrumentation. We determined the reproducibility of both configurations via a test–retest reliability experiment with 33 healthy subjects. The experiment confirmed that the proposals performed adequately, and allowed us to establish usage considerations. The MH-IMU configuration showed slightly less reproducibility than did the MH-OPT, but it still provided results that were equal to or even better than those in other studies. The MH system adds value to the field of gait analysis, and aims to improve the applicability of optical and IMU technologies in daily clinical practice. In this sense, if the MH system were used to conduct pre- and post-measurement sessions for the rehabilitation treatments or therapies applied, the MDC results would assist clinicians to assess the changes and to make better decisions for each individual patient.

## Figures and Tables

**Table 1:** Marker-to-marker distance (D) and maximum orientation error $\left(E_{o}\right)\;$ of each rigid body (RB). Positioning error in our camera configuration ($E_{p}=0.34\;\mathrm{mm})$.

Body Part	Side	$D_{1-2}[\mathrm{mm}]$	$D_{1-3}[\mathrm{mm}]$	$D_{2-3}[\mathrm{mm}]$	$E_{o}[{}^{\circ}]$	
Head	-	132.2	116.0	151.4	0.34	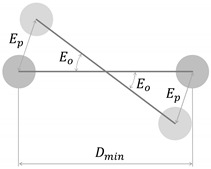 $E_{o}=2*\mathrm{arcsen}(\frac{E_{p}}{D_{min}})*\frac{180}{\pi }$
Arm	R	99.0	92.1	134.0	0.42	
Arm	L	113.7	91.6	117.1	0.43	
Forearm	R	104.4	81.3	95.6	0.48	
Forearm	L	116.3	75.3	85.7	0.52	
Hand	R	128.2	69.4	146.3	0.56	
Hand	L	70.3	120.3	135.6	0.55	
Chest	-	140.1	108.7	169.9	0.36	
Pelvic	-	180.6	105.3	163.0	0.37	
Thigh	R	126.2	114.8	99.6	0.39	
Thigh	L	95.4	105.2	122.3	0.41	
Calf	R	95.5	109.7	75.7	0.51	
Calf	L	126.3	70.1	82.4	0.56	
Foot	R	63.9	107.0	83.1	0.61	
Foot	L	114.0	64.1	94.1	0.61	
Mean (SD)	-	-	-	-	0.47 (0.09)	

R: right-hand side; L: left-hand side.

**Table 2:** Assumptions made for each body part to correct the magnetic north in the MH-IMU configuration.

IMU Sensor	Axis Projected on the Ground	Angle with the *Y*-Axis of the Pelvis Projected on the Ground [°]
Head	X	90
Arm	Y	90
Forearm	X	90
Hand	X	90
Chest	Y	0
Thigh	Y	90
Calf	Y	90
Foot	Y	0

The axes mentioned are those represented in Figure 3, considering that the participants had adopted the Fitbody position for the MH-IMU configuration represented in Figure 5.

**Table 3:** Variables considered in the study.

	Name	Description
**Spatio-temporal Variables** Dimensions that are based on whole-body movement	*Step length [cm]*	Distance between the centres of both ankle joints in the sagittal plane at T1.
	*Step width [cm]*	Distance between the centres of both ankle joints in the frontal plane at T1.
	*Single support [%]*	Percentage of mono-pedal support during the stride time. Percentage of T2 to T4 time with respect to the entire stride time.
	*Double support [%]*	Percentage of bipedal support during the stride time. Percentage of T1 to T2 time and T4 to T5 with respect to the entire stride time.
	*Gait speed [cm/s]*	Mean of the gait speed during the stride. Stride length divided by stride time.
**Kinematic Variables [°]** Dimensions that are based on the movement of each body segment	*Range of the trunk tilt. T2 to T5*	Chest rotation around the *Z*-axis with regard to the pelvic bone. Range from T2 to T5.
	*Range of the pelvic tilt. T1 to T4*	Pelvic bone rotation around the *Z*-axis. Range from T1 to T4.
	*Range of the hip flexion/extension. T1 to T4*	Hip joint rotation around the *X*-axis. Range from T1 to T4.
	*Range of the hip adduction/abduction. T4 to T5*	Hip joint rotation around the *Z*-axis. Range from T4 to T5.
	*Range of the knee flexion/extension. T4 to T5*	Knee joint rotation around the *X*-axis. Range from T4 to T5.
	*Range of the ankle dorsi/plantar flexion. T4 to T5*	Ankle joint rotation around the *X*-axis. Range from T4 to T5.
	*Range of the ankle inversion/eversion. T1 to T3*	Ankle joint rotation around the *Z*-axis. Range from T1 to T3.

**Table 4:** Test–retest reliability results.

		MH-IMU						MH-OPT					
		Test µ (SD)	Retest µ (SD)	Dif. µ (SD)	ICC	MCD es95	MCD 95	Test µ (SD)	Retest µ (SD)	Dif. µ (SD)	ICC	MCD es95	MCD 95
*Step length [cm]*	R	58.8 (4.6)	58.7 (4.8)	−0.1 (2.4)	0.93	0.8	3.5	59.5 (4.2)	59.5 (3.9)	0.0 (1.6)	0.96	0.5	2.3
	L	58.0 (5.0)	58.6 (5.0)	0.7 (2.1)	0.95	0.6	3.0	58.2 (4.0)	58.4 (4.3)	0.2 (1.4)	0.97	0.5	2.0
*Step width [cm]*	R	8.9 (3.5)	8.1 (4.0)	−0.8 (3.5)	0.72	1.6	5.5	12.8 (2.3)	12.6 (2.6)	−0.3 (1.2)	0.94	0.7	1.7
	L	10.1 (4.4)	9.9 (3.6)	−0.1 (4.1)	0.64	1.5	6.6	12.0 (2.1)	11.8 (2.5)	−0.2 (1.1)	0.94	0.8	1.6
*Single support [%]*	R	35.2 (2.3)	35.6 (2.2)	0.4 (1.1)	0.93	0.7	1.6	39.9 (0.9)	39.8 (0.9)	0.0 (0.5)	0.89	0.9	0.8
	L	36.0 (2.1)	36.4 (2.6)	0.4 (1.3)	0.91	0.9	1.9	39.8 (0.8)	39.7 (0.7)	0.0 (0.3)	0.95	0.6	0.5
*Double support [%]*	R	29.0 (3.9)	28.2 (4.2)	−0.8 (1.9)	0.94	0.7	2.7	20.4 (1.5)	20.4 (1.5)	0.1 (0.7)	0.95	0.6	1.0
	L	29.0 (4.0)	28.1 (4.4)	−0.8 (2.0)	0.94	0.7	2.8	20.4 (1.5)	20.4 (1.5)	0.0 (0.7)	0.95	0.6	0.9
*Gait speed [cm/s]*		121.8 (12.4)	123.3 (12.9)	0.1 (0.2)	0.97	0.5	6.5	114.2 (3.7)	114.3 (3.8)	0.0 (0.0)	1.00	0.2	0.7
*Range of trunk tilt T2 to T5 [°]*	R	10.0 (2.4)	9.8 (2.8)	−0.2 (1.2)	0.94	0.7	1.7	3.3 (2.1)	3.5 (1.9)	0.2 (1.4)	0.87	1.0	2.0
	L	10.0 (2.4)	9.8 (2.7)	−0.2 (1.2)	0.94	0.7	1.7	3.3 (2.1)	3.5 (1.9)	0.2 (1.3)	0.88	0.9	1.9
*Range of pelvic tilt T1 to T4 [°]*	R	4.8 (2.6)	4.6 (2.3)	−0.2 (1.1)	0.95	0.6	1.5	5.2 (2.1)	5.1 (2.0)	−0.1 (1.0)	0.94	0.7	1.4
	L	4.7 (2.5)	4.5 (2.3)	−0.2 (1.1)	0.95	0.6	1.5	5.2 (2.1)	5.1 (2.0)	−0.1 (1.0)	0.94	0.7	1.4
*Range of hip flexion-extension T1 to T4 [°]*	R	35.0 (3.5)	35.3 (4)	0.4 (1.9)	0.93	0.8	2.8	32.9 (3.3)	33.2 (3.6)	0.3 (1.6)	0.95	0.7	2.3
	L	35.3 (4.0)	35.6 (3.8)	0.3 (2.5)	0.89	0.9	3.6	33.4 (3.6)	33.1 (3.6)	−0.3 (1.8)	0.93	0.7	2.6
*Range of hip adduction-abduction T4 to T5 [°]*	R	7.7 (2.3)	7.7 (2.4)	0.0 (1.8)	0.83	1.2	2.7	5.1 (1.0)	5.2 (1.1)	0.2 (0.5)	0.93	0.8	0.8
	L	8.6 (2.4)	8.9 (2.8)	0.3 (1.9)	0.85	1.2	2.8	4.9 (1.0)	4.9 (1.1)	0.0 (0.6)	0.92	0.8	0.8
*Range of knee flexion-extension T4 to T5 [°]*	R	34.9 (6.2)	34.2 (7.1)	−0.8 (3.0)	0.95	0.7	4.2	22.4 (2.6)	22.9 (2.6)	0.5 (1.2)	0.94	0.7	1.7
	L	37.3 (6.4)	36.2 (6.5)	−1.0 (2.6)	0.96	0.6	3.7	22.0 (2.6)	22.2 (3.1)	0.1 (1.6)	0.91	0.9	2.4
*Range of ankle dorsi flexion. T4 to T5 [°]*	R	23.2 (4.0)	22.6 (4.0)	−0.6 (2.1)	0.92	0.8	3.1	15.6 (1.6)	15.4 (2.0)	−0.2 (1.6)	0.77	1.5	2.4
	L	22.6 (3.5)	23.2 (3.3)	0.5 (2.1)	0.89	0.9	3.1	14.9 (1.6)	15.1 (1.9)	0.2 (1.2)	0.87	1.1	1.7
*Range of ankle inv.-ev. T1 to T3 [°]*	R	8.1 (4.2)	9.5 (5.2)	1.4 (3.3)	0.86	1.1	4.8	9.1 (3.7)	9.3 (3.9)	0.2 (1.6)	0.95	0.6	2.3
	L	8.5 (3.9)	9.2 (4.3)	0.7 (3.0)	0.84	1.2	4.5	8.8 (3.8)	8.8 (4.2)	0.0 (1.3)	0.97	0.5	1.8

µ: Average; SD: Standard deviation; Dif.: Mean difference between a subject’s tests; ICC: Intraclass correlation coefficient; MDC95: Minimal detectable change at 95%; MDCes95: Effect size of the minimal detectable change at 95%; R: right-hand side; L: left-hand side.

**Table 5:** Comparison of the MDC results to those in other studies.

	MDCs in the Literature						MDCs in This Study		
	[93]	[94]	[95]	[96]	[97]		[92]	MH-IMU	MH OPT
**Spatio-temporal variables**									
*Step/Stride length [cm]*	8.0	8.0	-	4.0	5.4	11.0	10.0	3.2	2.2
*Step width [cm]*	3.0	2.0	-	2.0	2.3	-	-	6.0*	1.7
*Average of gait phases [%]*	1.9	-	-	-	-	1.5	1.7	2.3*	0.8
*Gait speed [cm/s]*	17.0	12.0	-	9.0	15.0	12.0	7.0	6.5	0.7
**Kinematic variables [°]**									
*Range of trunk tilt*	-	2.5	1.1	-	-	-	-	1.7	2.0*
*Range of pelvic tilt*	1.9	4.4	2.5	-	-	-	-	1.5	1.4
*Range of hip flexion/extension*	4.4	8.3	2.7	3.0	3.3	-	-	3.2	2.4
*Range of hip adduction/abduction*	3.0	5.1	2.6	2.0	5.5	-	-	2.8	0.8
*Range of knee flexion/extension*	4.0	4.5	5.1	3.0	3.5	-	-	4.0	2.1
*Range of ankle dorsi/plantar flexion*	8.7	4.1	3.5	-	8.5	-	-	3. 1	2.1
*Range of ankle inversion eversion*	-	9.6	-	-	-	-	-	4.7	2.0
**Study details**									
*Experimental conditions*	Floor	Floor	Floor	Treadmill	Treadmill	Floor	Treadmill	Floor	Treadmill
*MoCap technology*	Full body optical	Full body optical	Full body optical	Full body optical	Lower body optical	Feet placement IMU		Full body IMU	Full body optical
*Sample*	30 (18F, 12M)	23 (12F, 11M)	29 (15F, 14M)	20 (10F, 10M)	23 (23M)	39 (14F, 25M)		33 (13F, 20M)	
*Age*	30 ± 6.8	35 ± 7.3	24 ± 5.7	25 ± 4.0	35 ± 5.1	23 ± 6.2		22 ± 2.9	
*Rater*	Same	Same	Same	Same	Same (except for anatomical measures)	Not found		Same	
*Time*	1 to 14 days	One week	5.6 ± 2.2 days	One week	5 ± 3.0 days	More than one day		Three hours	
*Notes*	Gait phases averaged: Foot off, Opposite foot contact, and Opposite foot off. MDC from stride length.	They do not provide ranges of movement. We have averaged the MDC of the peaks. MDC from stride length.	Data from ‘intrarater intersession’ at ‘FR3’ speed.	Some ranges are from max. to min. and others from one gait event to another. MDC from step length.	Right and left sides averaged. MDC from step length.	Gait phases averaged: Stance and Swing. Feet IMU placement selected; this was preferred by the authors. MDC from stride length.		Gait phases averaged: Single and double support. Right and left sides averaged. The ranges are from one gait event to another. MDC from step length.	

* Values with worse MDCs than the average in other studies.
